# Editorial: Tuberous Sclerosis Complex – Diagnosis and Management

**DOI:** 10.3389/fneur.2021.755868

**Published:** 2021-10-05

**Authors:** Sergiusz Jozwiak, Paolo Curatolo

**Affiliations:** ^1^Department of Child Neurology, Medical University of Warsaw, Warsaw, Poland; ^2^Department of System Medicine, University of Rome Tor Vergata, Rome, Italy

**Keywords:** tuberous sclerosis complex (TSC), diagnosis, treatment, epilepsy, genetics

Tuberous sclerosis complex (TSC) is a multisystem genetic disorder caused by a mutation in either the TSC1 or TSC2 gene and resulting in an overactivation of the mTOR pathway that affects many organs and systems (1).

Though it has been 150 years since the first clinically reported case of tuberous sclerosis, there are still many gaps in our understanding of its pathogenesis, clinical symptomatology, comorbidities, prognostic significance, genetic heterogeneity, and therapeutic possibilities. Moreover, as it is a rare disorder, TSC faces all the difficulties that are characteristic of small cohorts of patients. The creation of a multicentered, multinational TuberOus SClerosis registry to increase disease awareness (TOSCA registry), consisting of 2,221 TSC patients from 31 countries, was intended to address these gaps. Data collected on the natural history of TSC, the incidence of the TSC manifestations, and the age at their onset, are included in several papers published in this Special Issue.

Subependymal giant-cell astrocytomas (SEGAs) are among the most frequent and life-threatening manifestations of TSC. Two papers by Jansen, Belousova, Benedik, Carter, Cottin, Curatolo, Dahlin et al. report the natural course and treatment characteristics in 554 patients from 2,216 TOSCA participants (25%). The median age at diagnosis of SEGAs was 8 years (range, 0–51). SEGAs were symptomatic in 42.1% of patients. Symptoms included increased seizure frequency (15.8%), behavioral disturbance (11.9%), and regression/loss of cognitive skills (9.9%). SEGAs were significantly more frequent in patients with *TSC2* compared to *TSC1* variants (33.7 vs. 13.2%, *p* < 0.0001). Interestingly, mammalian targets of rapamycin inhibitors (mTORi) were almost as frequently used as surgery (49 vs. 59.6%). However, this finding may be biased in the TOSCA study by the high proportion of sites participating in EXIST-1 trial.

Another important finding from the Jansen, Belousova, Benedik, Carter, Cottin, Curatolo, Dahlin, D'Amato et al. paper is the possibility of SEGA development in adult patients. Fourteen adults (2.4%) were newly diagnosed with SEGAs during follow-up, and all had the *TSC2* mutation.

Renal angiomyolipomas (AMLs) are one of the most common renal manifestations in patients with TSC, with potentially life-threatening complications and a poor prognosis. In the study of Kingswood, Belousova, Benedik, Carter, et al., they were significantly more prevalent in female patients (*p* < 0.0001). Although renal AMLs in subjects with *TSC1* mutations develop on average at a later age and are relatively smaller, by age 40 no difference was observed in the percentage of patients with *TSC1* and *TSC2* mutations needing intervention.

Non-functional pancreatic neuroendocrine tumors (PNETs) are rare in TSC, with no specific guidelines outlined for clinical management. Mowrey et al. reported 16 individuals, nine males and seven females with an average age of 22.0 years, and characterized the course of the tumors and applied management. The calculated prevalence of non-functional PNETs in their group was 0.65%.

Individuals with TSC are at increased risk of developing both epilepsy and autism. mTOR dysregulation could play a direct role in determining susceptibility to epilepsy, cognitive impairment, and autism spectrum disorder (2). The identification of early signs may therefore become an important prevention tool.

In two papers by Nabbout et al. epilepsy appeared in 85% of patients with a high incidence of drug-resistant epilepsy. *TSC1* mutations were associated with less severe epilepsy phenotypes and more individuals with normal IQ. GABAergics were the first-line treatment in 45% of children with infantile spasms. Prenatal or early infantile diagnosis of TSC provides a unique opportunity to monitor EEG before the onset of clinical seizures. Recently, the EPISTOP trial provided crucial information on the optimal timing for initiating treatment in high-risk infants (3).

A relatively low percentage (12.5–25%) of patients with EEG performed before seizures in the paper by Nabbout et al. may be partly explained by the retrospective character of the study and the large proportion of currently adult patients in the TOSCA registry.

A better understanding of the early biomarkers of developmental outcome could give us a therapeutic window in which early-targeted treatment could obtain greater benefits. De Ridder et al. investigated whether early EEG characteristics in newborns and infants with TSC can be used to predict neurodevelopment. The first recorded EEG of 64 infants with TSC, enrolled in the international prospective EPISTOP trial was correlated with ASD risk based on the ADOS-2 score, and cognitive, language, and motor developmental quotients (Bayley Scales of Infant and Toddler Development III) at the age of 24 months. A dysmature EEG background was associated with lower cognitive, language, and motor developmental quotients at the age of 24 months indicating that early EEG characteristics can be used to predict neurodevelopmental comorbidities in infants with TSC.

The complex relationship between epilepsy and comorbid autism in TSC has been discussed by Specchio et al.. They highlighted the need for early identification and management to optimize favorable outcomes in infants at high risk of developing early seizure onset and autism.

Prenatal or early infantile diagnosis of TSC provides a unique opportunity to monitor EEG before the seizure onset (4). The prevention of epilepsy in infants with TSC is becoming an important challenge. Recently the EPISTOP trial demonstrated that preventive treatment with Vigabatrin reduced the risk and severity of epilepsy (3). Early developmental markers of ASD, such as a social communication deficit, may be identified in the first year of life (5), allowing early intervention in infants at high risk of developing autism, with the potential of optimizing developmental outcomes.

In a *post-hoc* analysis focused on pediatric patients enrolled in the EXIST3 trial, adjunctive everolimus resulted in a sustained reduction in seizure frequency, with particular efficacy in younger children under the age of 6 years (6). Preventive trials with mTOR inhibitors could now be designed in pre-symptomatic infants to evaluate if this strategy could have disease-modifying effects.

Everolimus, a disease-modifying drug that targets the molecular biology of TSC, can address multiple aspects of the disease at the same time. Kingswood, Belousova, Benedik, Budde et al. assessed the long-term safety of everolimus in a non-interventional post-authorization safety study (PASS) in patients with TSC who participated in the TOSCA clinical study and received everolimus for the licensed indications in the European Union. One hundred and eighteen of 179 (66%) patients had an adverse effect (AE) of any grade, with the most common AEs being stomatitis (7.8%) and headache (7.3%). AEs caused dose adjustments in 31.3% and treatment discontinuation in 5% of patients.

To achieve beneficial, suppressing effects of mTORi on growing tumors in TSC, persistent drug treatment is necessary. The aim of EMINENTS prospective, single-center, open-label, single-arm study was to evaluate the cumulative efficacy and safety of reduced doses of everolimus (maintenance therapy) in patients TSC and SEGA. Bobeff et al. included 15 patients who had undergone at least 12 months of treatment with a standard everolimus dose. The dose of everolimus was reduced to three times a week, and patients were followed over a mean duration of 58.37 months. No clinical symptoms of progression were observed in any patients. Regarding AEs, infections and laboratory abnormalities occurred less frequently during maintenance therapy compared to the standard dose regimen.

Marques, Belousova, Benedik, Carter, Cottin, Curatolo, Dahlin, D'Amato, d'Augères, de Vries, Ferreira, Feucht, Fladrowski, Hertzberg, Jansen et al. showed evidence that the TOSCA registry improved the knowledge on the natural history and manifestations of TSC, increased awareness, produced real-world evidence, and helped to identify relevant information for future clinical research.

The authors provided a comprehensive picture of the medical and non-medical health care resources in TSC from information within the TOSCA registry. GABAergic were the most prescribed drugs for epilepsy, and mTOR inhibitors were dramatically replacing surgery in patients with SEGA, despite current recommendations proposing both treatment options (Marques, Belousova, Benedik, Carter, Cottin, Curatolo, Dahlin, D'Amato, d'Augères, de Vries, Ferreira, Feucht, Fladrowski, Hertzberg, Jozwiak et al.).

In another paper, Marques, Thole et al. tested the recommendations from the European Medicine Agency (EMA) on the rare disease registries. They elaborated the compliance and deviations of the TOSCA registry from the EMA guidance on a point-by-point basis, revealing that in most aspects the TOSCA registry met its objective to enhance our understanding of TSC and its manifestations.

Research on TSC to date has focused mainly on the physical manifestations of the disease. One study in this issue examines the psychosocial impact of TSC, which has received until now less attention. Jansen, Vanclooster et al. investigated the quality of life from the TOSCA study and highlighted the substantial burden of the disease on the personal lives of individuals with TSC. A smooth transition from pediatric to adult care was mentioned by only 36% of caregivers.

TAND poses significant challenges for the diagnosis and management of TSC. Although knowledge is increasing about TAND, little is known about the confounding effects of intellectual ability and the rate of TAND across age, sex, and genotype. de Vries et al. demonstrated in this issue that there is a significant association between levels of intellectual ability and the majority of TAND manifestations. However, no significant age or sex differences were observed from academic difficulties or neuropsychological deficits.

Current recommendations for the delivery of services for TSC patients, including diagnosis, surveillance, treatment, and safe transition from pediatric to adult care, are the focus of Annear et al.. TSC clinics need to offer a range of core services in order to provide comprehensive treatment to TSC patients. Furthermore, TSC clinics should have a multidisciplinary team with a dedicated specialist TSC coordinator, with the aim of ensuring that each TSC patient and their family have a tailored care plan to manage current manifestations and surveillance for future disease manifestations.

In this special issue, several papers were related to TOSCA to expand the current knowledge on diagnosis and management of TSC, allowing the broadening of preventive strategies and stimulating further research in the field. There is now the need to improve TSC management to ensure patients have early access to appropriate treatment and preventive measures.

## Author Contributions

SJ and PC worked equally on editorships, reviewing the articles, and writing the editorial. Both authors contributed to the article and approved the submitted version.

## Conflict of Interest

The authors declare that the research was conducted in the absence of any commercial or financial relationships that could be construed as a potential conflict of interest.

## Publisher's Note

All claims expressed in this article are solely those of the authors and do not necessarily represent those of their affiliated organizations, or those of the publisher, the editors and the reviewers. Any product that may be evaluated in this article, or claim that may be made by its manufacturer, is not guaranteed or endorsed by the publisher.
